# Supplementary material for: Water-Activated Semiquinone Formation and Carboxylic Acid Dissociation in Melanin Revealed by Infrared Spectroscopy
Source: Polymers (Basel). 2021 Dec 15;13(24):4403. doi: 10.3390/polym13244403 (PMC8705668; doi:10.3390/polym13244403)
Supplement: Supplementary file 1 [file polymers-13-04403-s001.zip › polymers-1462568-supplementary.pdf]

Supplementary Materials

Water-Activated Semiquinone Formation and Carboxylic Acid Dissociation in Melanin Revealed by Infrared Spectroscopy

Zakhar V. Bedran [1], Sergey S. Zhukov [1], Pavel A. Abramov [1], Ilya O. Tyurenkov [1], Boris P. Gorshunov [1], A. Bernardus Mostert [2] and Konstantin A. Motovilov [1,*]

[1] Center for Photonics and 2D Materials, Moscow Institute of Physics and Technology, Institute Lane 9, 141701 Dolgoprudny, Russia; bedran@phystech.edu (Z.V.B.); zs1978@mail.ru (S.S.Z.); abramovpa33@gmail.com (P.A.A.); turenkov1997@mail.ru (I.O.T.); bpgorshunov@gmail.com (B.P.G.)

[2] Department of Chemistry, Swansea University, Singleton Park, Swansea SA2 8PP, Wales, UK; a.b.mostert@swansea.ac.uk

* Correspondence: k.a.motovilov@gmail.com

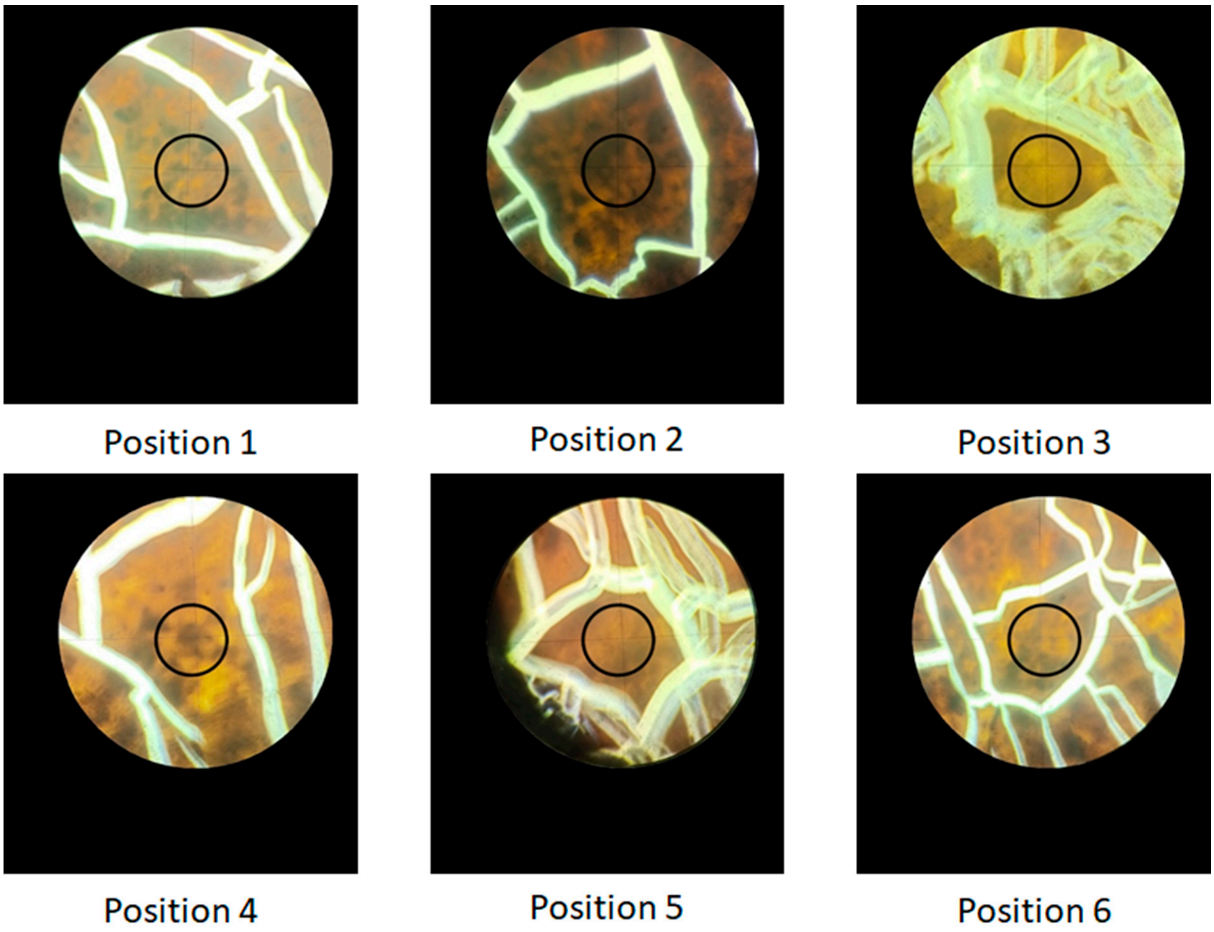

Figure S1. The microscope images of the six positions on synthetic eumelanin thin film. The black circles in the middle of each tile is the area exposed to an IR radiation beam with a 50 µm diameter.

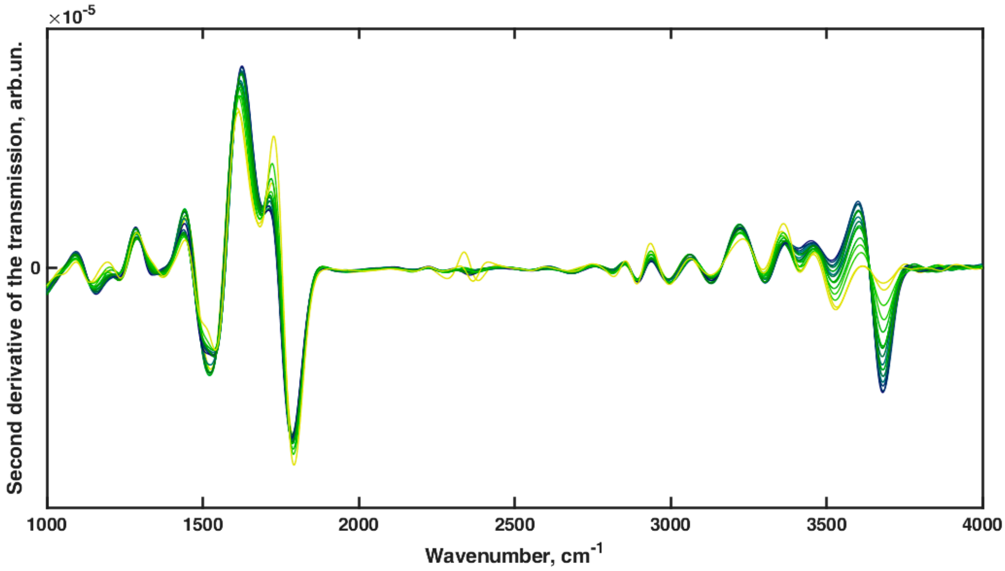

Figure S2.1. The observed evolution of the second derivative of the transmission spectra with the reversible RH variation. α = 50. Position = 1.

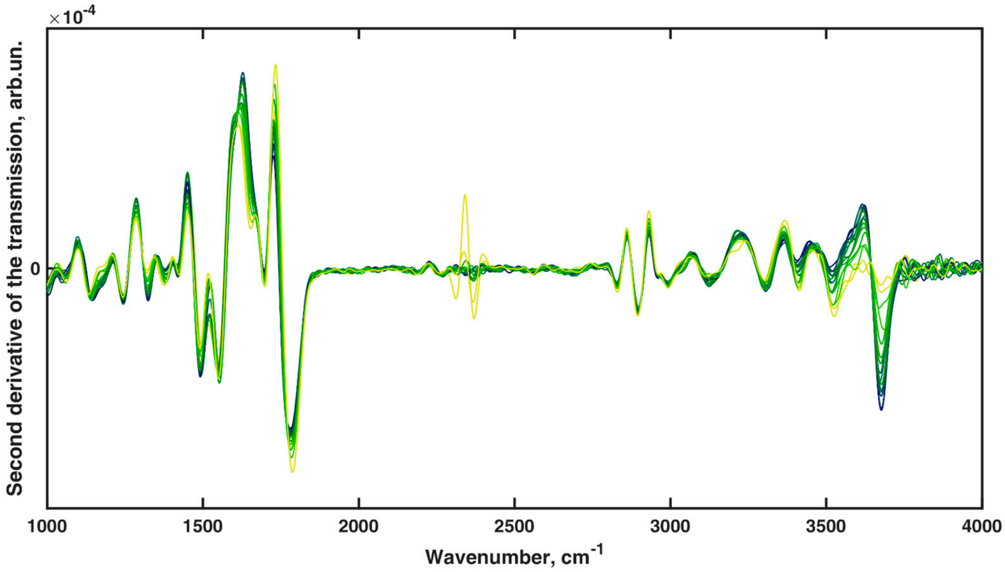

Figure S2.2. The observed evolution of the second derivative of the transmission spectra with the reversible RH variation. α = 5. Position = 1.

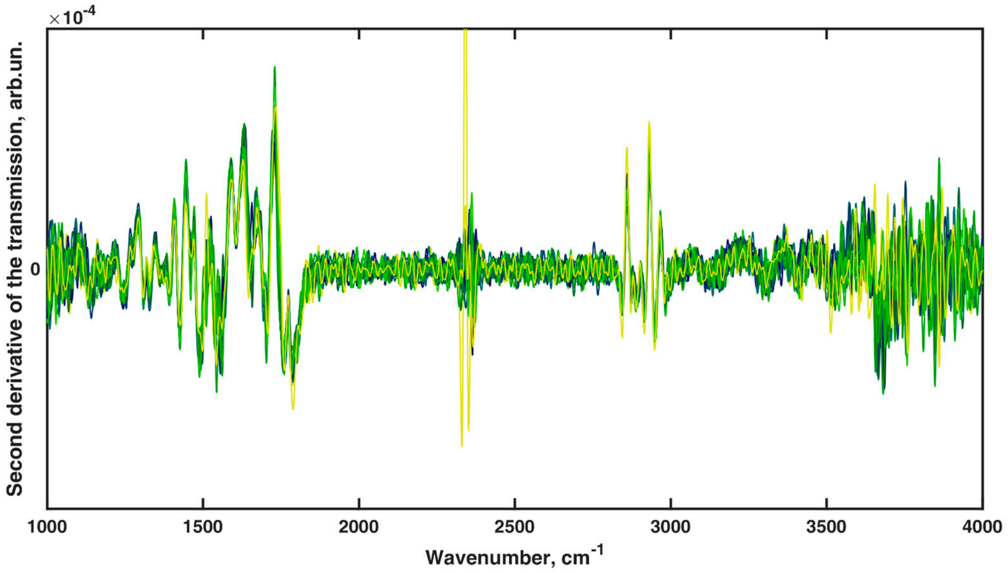

Figure S2.3. The observed evolution of the second derivative of the transmission spectra with the reversible RH variation. α = 0.1. Position = 1.

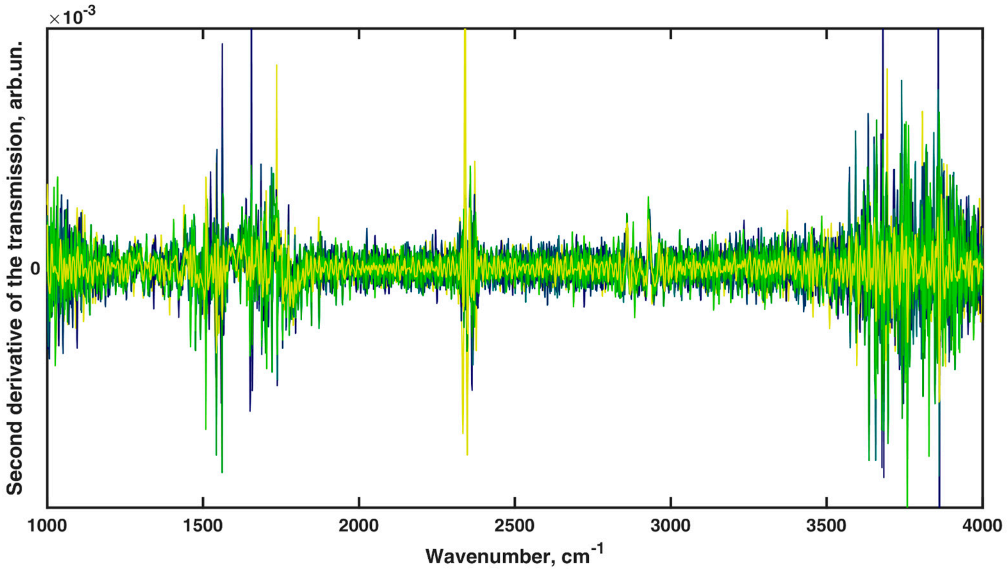

Figure S2.4. The observed evolution of the second derivative of the transmission spectra with the reversible RH variation. α = 0.01. Position = 1.

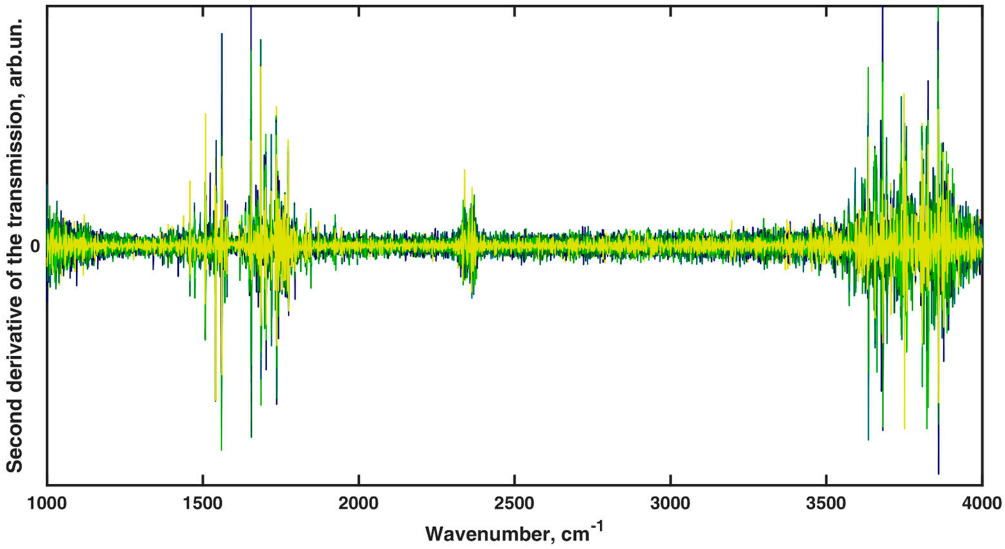

Figure S2.5. The observed evolution of the second derivative of the transmission spectra with the reversible RH variation. α = 0.001. Position = 1.

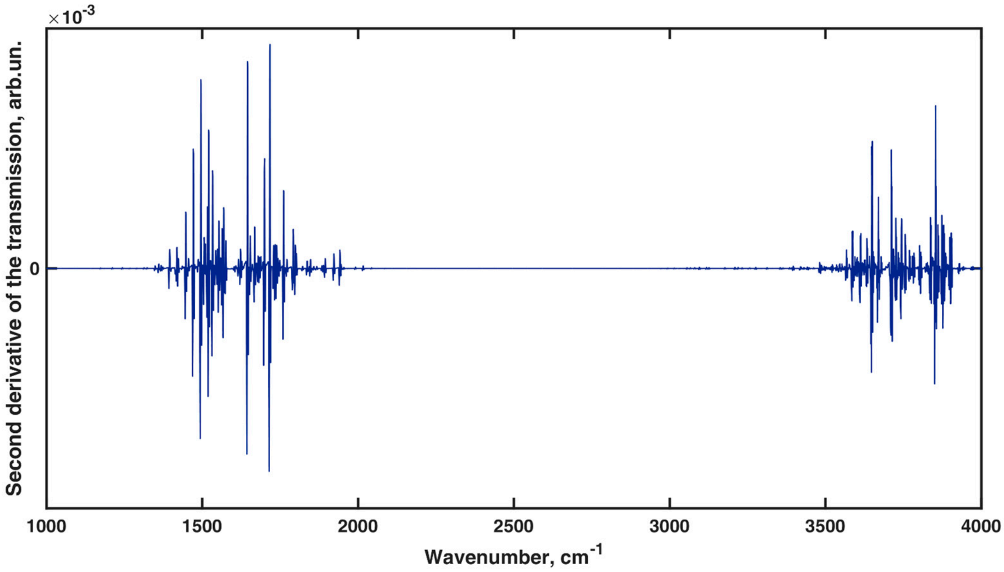

Figure S2.6. The second derivative of the transmission spectra of water vapor. The data simulated from HITRAN database. α = 0.001.

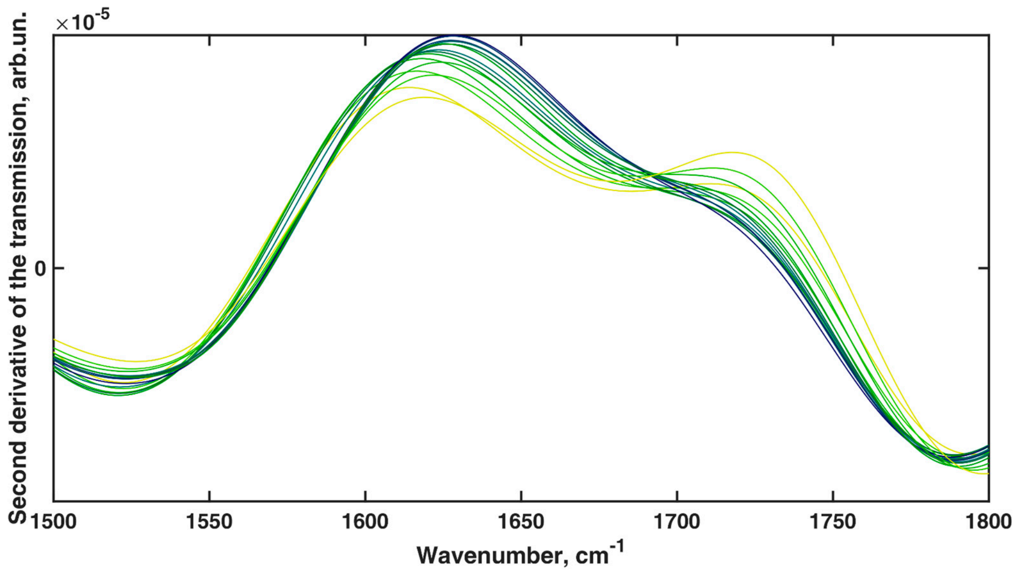

Figure S3.1. The demonstration of the RH-dependent second derivative of the transmission spectra dependence from α. α = 100.

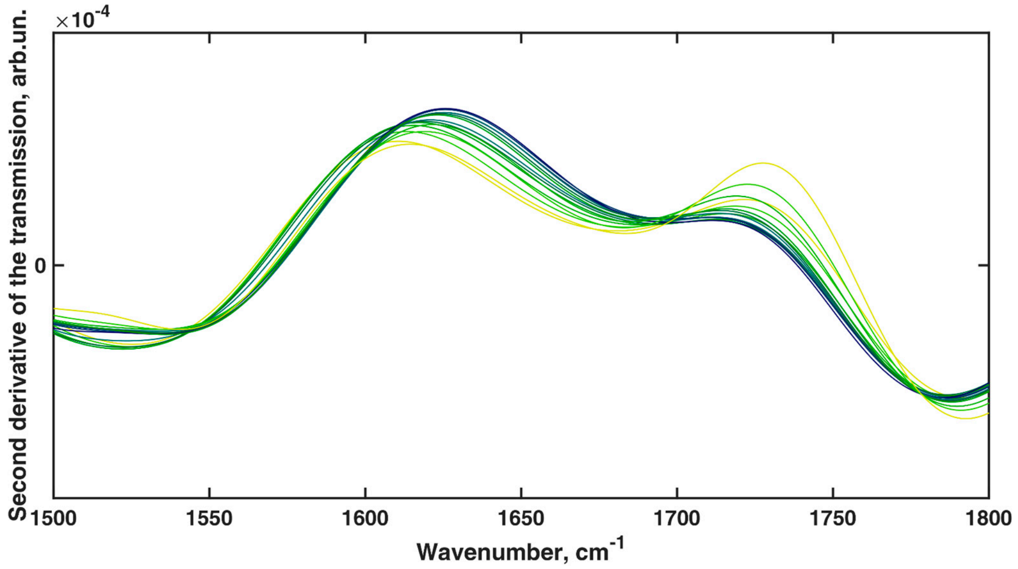

Figure S3.2. The demonstration of the RH-dependent second derivative of the transmission spectra dependence from α. α = 50.

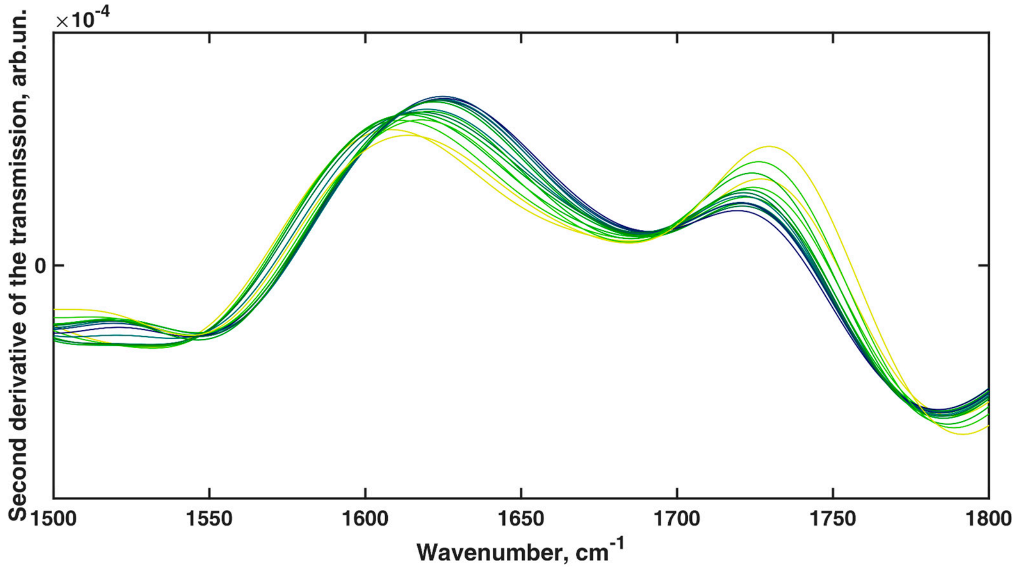

Figure S3.3. The demonstration of the RH-dependent second derivative of the transmission spectra dependence from α. α = 25.

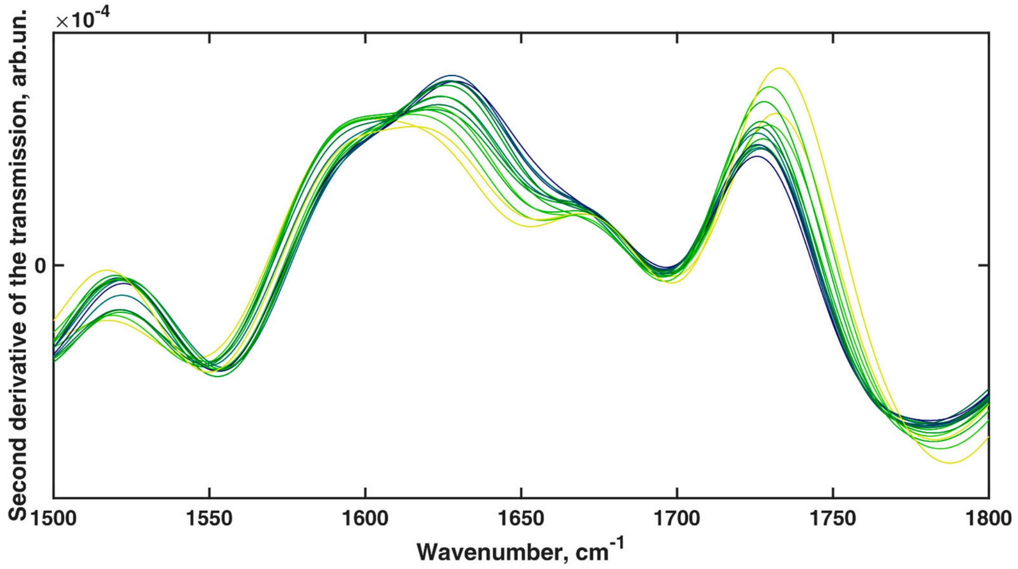

Figure S3.4. The demonstration of the RH-dependent second derivative of the transmission spectra dependence from α. α = 5.

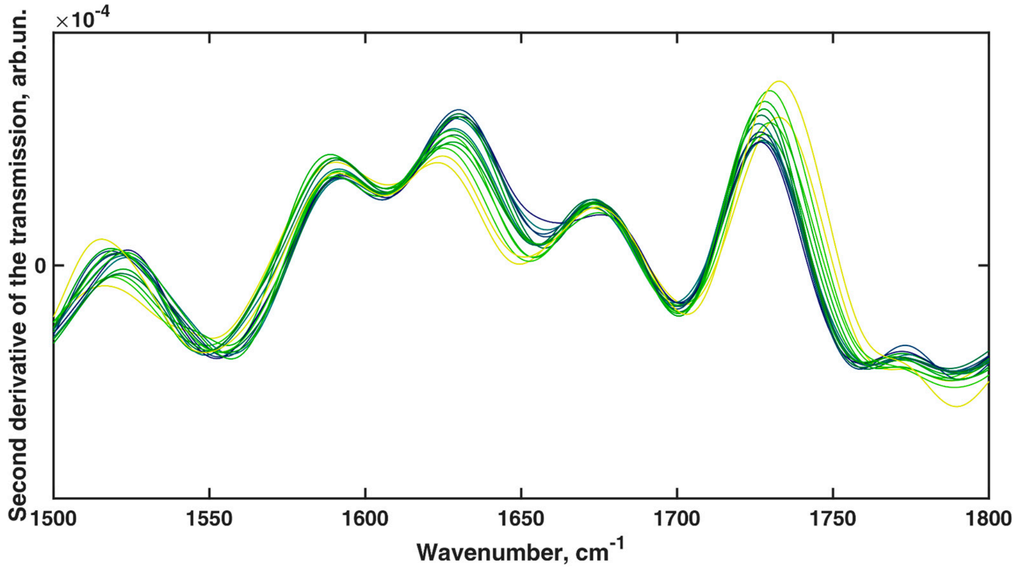

Figure S3.5. The demonstration of the RH-dependent second derivative of the transmission spectra dependence from α. α = 1.

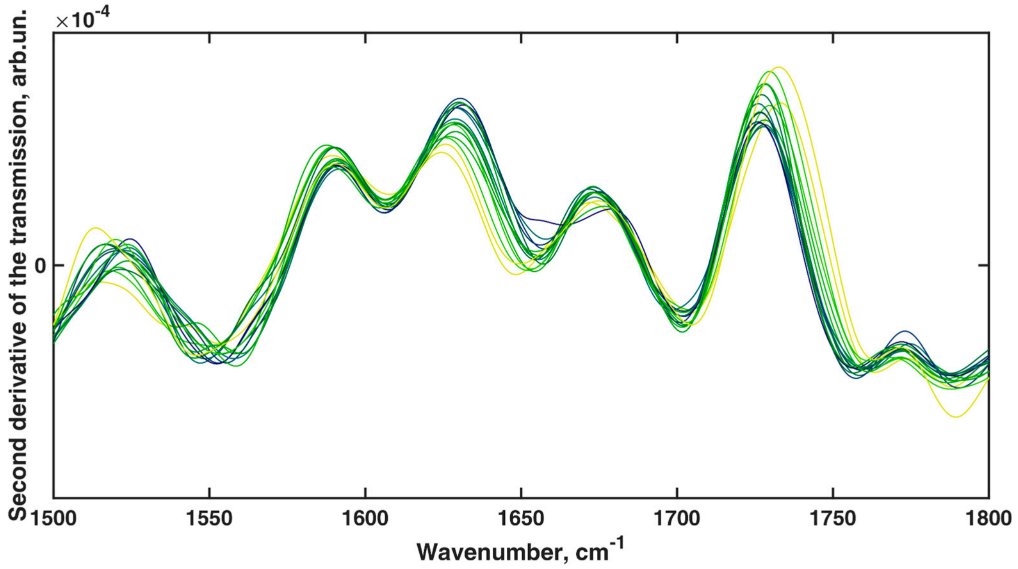

Figure S3.6. The demonstration of the RH-dependent second derivative of the transmission spectra dependence from α. α = 0.5, position №1.

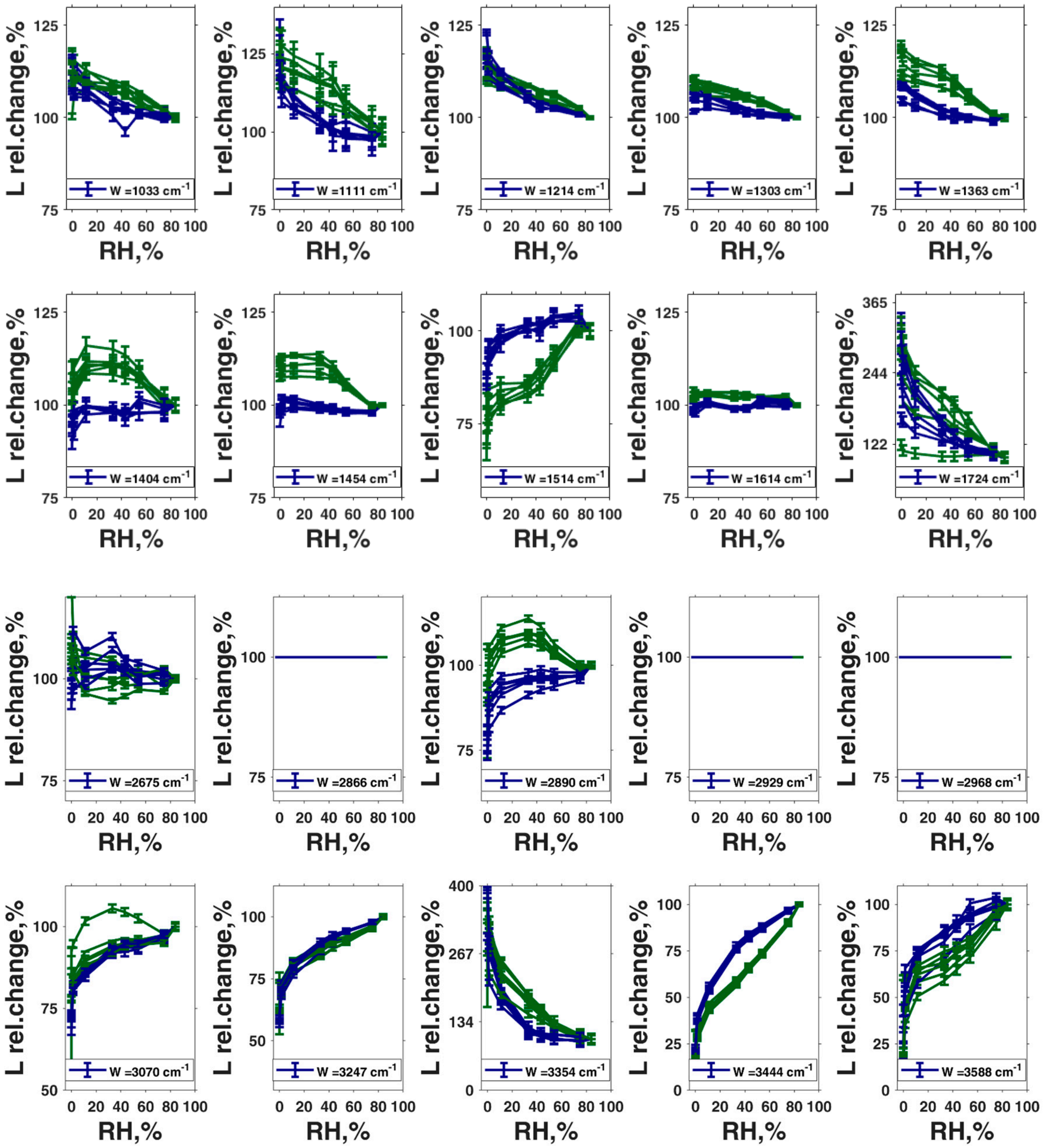

Figure S4. - 84%. The observed evolution of the relative line strength with hydration of all detected modes. In the legend, W refers to the central wavenumber of the mode in cm^{-1}, L at 84% RH is normalization for this mode. The error bars were estimated from the L-M LSDM algorithm. The green lines correspond to changes in humidity from 1.4% RH to 84% RH, and blue lines, respectively, from 75% RH to 0% RH.

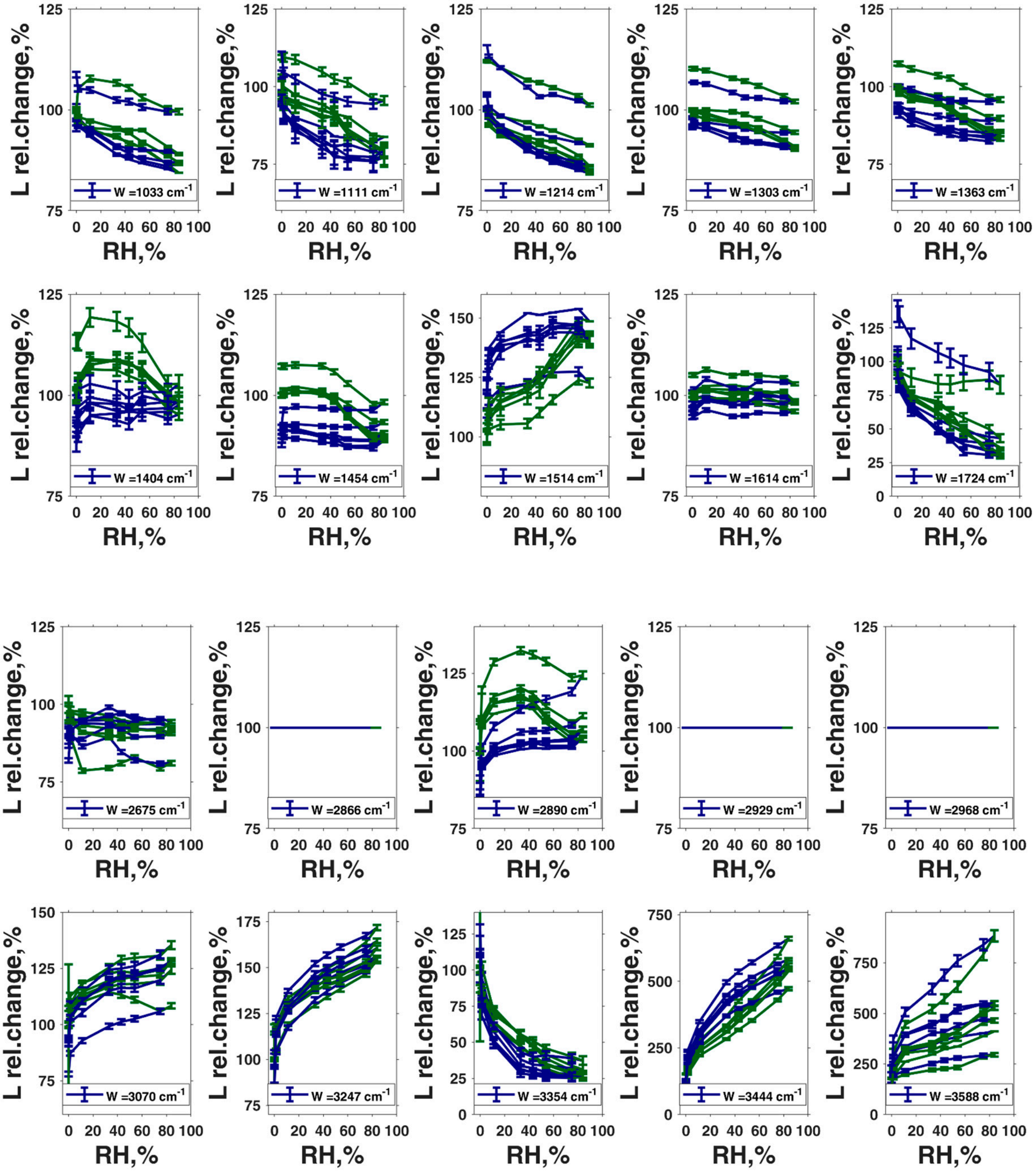

Figure S5. - 0%. The observed evolution of the relative line strength with hydration of all detected modes with reversible moisture change. On the legend, W refers to the central wavenumber of the mode in cm^{-1}, L at 1.4% RH is normalization for this mode. The error bars were estimated from the L-M LSDM algorithm. The green lines correspond to changes in humidity from 1.4% RH to 84% RH, and blue lines, respectively, from 75% RH to 0% RH.
